# Effect of MWCNT size, carboxylation, and purification on in vitro and in vivo toxicity, inflammation and lung pathology

**DOI:** 10.1186/1743-8977-10-57

**Published:** 2013-11-13

**Authors:** Raymond F Hamilton, Zheqiong Wu, Somenath Mitra, Pamela K Shaw, Andrij Holian

**Affiliations:** 1 Department of Biomedical and Pharmaceutical Sciences, Center for Environmental Health Sciences, University of Montana, Missoula, MT 59812, USA; 2 Department of Chemistry and Environmental Science, New Jersey Institute of Technology, Newark, NJ 07102, USA

**Keywords:** MWCNT, Macrophage, NLRP3 inflammasome, Functionalization, Nanoparticles

## Abstract

**Background:**

Several properties of multi-walled carbon nanotubes (MWCNT) have the potential to affect their bioactivity. This study examined the *in vitro* and *in vivo* outcomes of the influence of diameter, length, purification and carboxylation (*in vitro* testing only) of MWCNT.

**Methods:**

Three original ‘as received’ MWCNT that varied in size (diameter and length) were purified and functionalized by carboxylation. The resulting MWCNT were characterized and examined for cytotoxicity and inflammasome activation *in vitro* using THP-1 cells and primary alveolar macrophages from C57BL/6 mice. Oropharyngeal aspiration administration was used to deliver original MWCNT and *in vivo* bioactivity and lung retention was examined at 1 and 7 days.

**Results:**

Studies with THP-1 macrophages demonstrated that increased length or diameter corresponded with increased bioactivity as measured by inflammasome activation. Purification had little effect on the original MWCNT, and functionalization completely eliminated bioactivity. Similar results were obtained using alveolar macrophages isolated from C57BL/6 mice. The *in vivo* studies demonstrated that all three original MWCNT caused similar neutrophil influx at one day, but increasing length or diameter resulted in the lavaged cells to release more inflammatory cytokines (IL-6, TNF-α, and IL-1β) *ex vivo*. Seven-day histology revealed that, consistent with the *in vitro* results, increasing width or length of MWCNT caused more severe pathology with the longest MWCNT causing the most severe inflammation. In addition, the same two larger MWCNT were retained more in the lung at 7 days.

**Conclusions:**

Taken together, the results indicated that *in vitro* and *in vivo* bioactivity of MWCNT increased with diameter and length. Purification had no significant modifying effect from the original MWCNT. Functionalization by carboxylation completely eliminated the bioactive potential of the MWCNT regardless of size in *in vitro* testing.

## Background

Engineered carbon nanomaterials such as multi-walled carbon nanotubes (MWCNT) have applications in structural and electronic devices due to their extraordinary thermal conductivity, mechanical and electrical properties, which creates a potential occupational exposure situation [1]. Potential bioactivity (*in vitro* toxicity and increased production of inflammatory mediators, and/or *in vivo* increased inflammation and pathology) of MWCNT has been attributed to length [2,3], diameter [4], aggregation state [5], contaminants [6-8], aspect ratio/rigidity [3,9], and release of reactive oxygen species [10]. Surface modification of MWCNT with functional moieties is an important step in creating useful biological and industrial nanomaterials [11,12]. Surface functional groups can alter the surface charge, functionality and reactivity of the surface, and enhance the stability, and dispersability of MWCNT [13,14]. Raw MWCNT from commercial vendors usually contain metal impurities (e.g., Ni, Fe) and a surface amorphous carbon layer. The presence of metal impurities and the surface amorphous carbon jeopardizes the intrinsic optical, electrical and mechanical properties of MWCNT and can have undesirable biological activities [15,16].

Due to their unique physical and chemical characteristics, MWCNT may have distinct biological effects when inhaled [2,17,18]. Several studies have focused on particle retention in the lung, linked to MWCNT length or rigidity as a potential area of concern [2,9,19]. Still another study cites MWCNT diameter as the particle property that could affect *in vivo* bioactivity [4]. Nevertheless, it is apparent that MWCNT size is a potential critical factor in lung pathology.

For nanoparticle interaction at the cellular level, a number of studies have linked phago-lysosomal permeablization accompanied by cathepsin B release, which initiates NLRP3 inflammasome assembly and Caspase-1 activation as critical steps in particulate-induced inflammation [8,20-23]. While recent studies have suggested that toxic nanomaterials can initiate this lysosomal damage leading to NLRP3 inflammasome activation, the exact mechanisms are unknown at this time [3,24]. The *in vitro* endpoints used in this study, toxicity and NLRP3 inflammasome activation (using IL-1β as a proxy biomarker measure), were designed to explore the contribution of MWCNT properties, such as size, and surface modification on bioactivity. The *in vivo* endpoints were designed to look at initial inflammation and resulting pathology linked to particle retention and bioactivity.

The hypothesis in this study is that the width and length of MWCNT are important determinants in MWCNT bioactivity both *in vitro* and *in vivo*. Also, surface modifications in the form of purification and adding carboxyl groups to the surface of MWCNT, are proposed to alter or eliminate the bioactivity of the MWCNT in macrophages exposed *in vitro*. In the current study, *in vitro* assessments of the bioactivity of the MWCNT were conducted using primary alveolar macrophages (AM) isolated from C57Bl/6 mice and differentiated THP-1 cells with respect to toxicity and activation of the NLRP3 inflammasome. *In vivo* studies used C57BL/6 mice instilled with the original MWCNT particles only, and were examined one and seven days later.

## Results

### Particle Characterization

**Figure 1:**
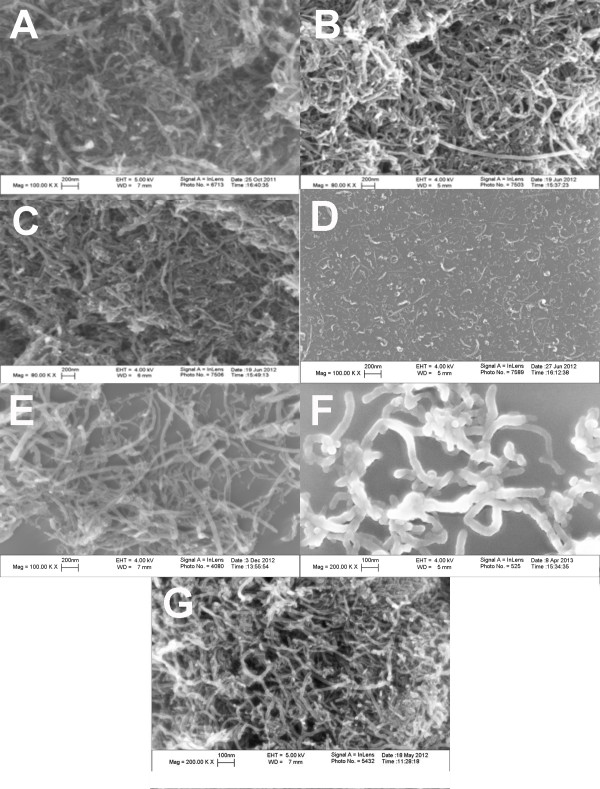
**SEM images of MWCNT used in this study. A**) narrow/short-original (N/S-O) MWCNT, **B**) narrow/short-functionalized (N/S-F) MWCNT, **C**) narrow/short-purified (N/S-P) MWCNT. Length comparison shows in **D**) narrow/short-functionalized (N/S-F) MWCNT, and **E**) narrow/long-functionalized (N/L-F) MWCNT. Diameter comparison shows in **F**) wide/short-functionalized (W/S-F) MWCNT, and **G**) narrow/long-functionalized (N/L-F) MWCNT.

The main metal impurities in MWCNT-O samples were iron and nickel, and the concentrations in the various samples are presented in Table 1. Iron was removed by both functionalization and purification. The content of nickel decreased after purification, while nickel could only be totally removed by functionalization. Elemental analysis (Table 1) showed the percentage of oxygen in MWCNT-F was much higher than in the corresponding MWCNT-O, which was due to the generation of carboxylic groups. SEM images of raw, purified and functionalized MWCNT for the narrow/short are shown in Figure 1A, C. These images show that the MWCNT remained intact with minimal visible tube damage after purification and functionalization. Figure 1D, E showed the obvious length difference between (N/S-F) MWCNT and (N/L-F) MWCNT while Figure 1F, G illustrates the significant diameter difference between (W/S-F) MWCNT and (N/L-F) MWCNT. Surface areas were measured by standard BET for the three original MWCNT (Table 1). The N/S-O had the lowest at 141 m^2^/g, while the N/L-O was approximately 50% greater at 217 m^2^/g and the W/S-O was slightly less than the N/L-O at 205 m^2^/g.

**Table 1:** ***Elemental analysis*, length, diameter and surface area of MWCNT**

**MWCNT**	**C % by**	**O % by**	**Fe % by**	**Ni % by**	**Diameter**	**Length**	**Surface area**
	**weight**	**weight**	**weight**	**weight**	**(nm)**	**(nm)**	**(m**^**2**^**/g)**
N/S-O	96.30	1.66	0.85	1.19	17.78 ± 3.79	1108.45 ± 459.81	140.6
N/S-F	90.59	9.41	-	-	24.46 ± 6.36	897.67 ± 430.49	nd
N/S-P	98.23	0.92	-	0.85	18.87 ± 5.10	1007.03 ± 399.77	nd
W/S-O	95.53	3.49	0.51	0.47	31.67 ± 6.52	1198.58 ± 353.06	204.9
W/S-F	86.28	13.72	-	-	32.50 ± 9.01	760.92 ± 518.89	nd
W/S-P	97.18	2.42	-	0.40	30.19 ± 7.64	810.79 ± 463.81	nd
N/L-O	98.46	0.72	-	0.82	15.49 ± 3.39	-*	217.3
N/L-F	86.74	13.26	-	-	22.69 ± 5.39	-*	nd
N/L-P	99.17	0.37	-	0.46	16.72 ± 3.56	-*	nd

* impossible to obtain accurate lengths due to excessive tangling.

nd – not determined.

N/S: narrow diameter/short length; W/S: wide diameter/short length; N/L: narrow diameter/long length.

O: original MW; F: functionalized MW; P: purified MW.

**Figure 2:**
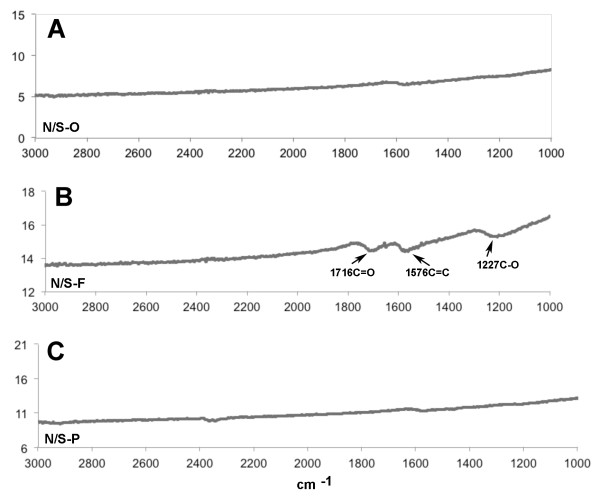
**FTIR Data for narrow/short MWCNT variants. A**) N/S-O MWCNT, **B**) N/S-F MWCNT, and **C**) N/S-P MWCNT.

The FTIR spectra in Figure 2A, C show evidence of functionalization via carboxylation. The carboxylic stretching frequency (C = O) at 1716 cm^-1^ and stretching vibration (C-O) at 1227 cm^-1^, provided evidence of carboxyl groups in all MWCNT-F samples (Figure 2B), which was absent in MWCNT-O or MWCNT-P (Figure 2A and 2C respectively). C = C stretching at 1576 cm^-1^, which exists in the carbon skeleton, was present in all materials. The ratio between free carbon and carboxylated carbon and length, diameter data are also shown in Table 1. The functional groups attached to the sidewall and the end of MWCNT-F led to an apparent increase in diameter (Table 1). This was not observed for MWCNT-P. Furthermore, in the FTIR, N/L-O and W/S- O showed similar functional group peaks as N/S-O; N/L-P and W/S- P showed similar functional group peaks as N/S-P; while N/L-F and W/S- F showed similar functional group peaks as N/S-F.

Dynamic light scattering (DLS) was used to measure size of particle agglomerates in suspensions of all samples (Table 2). MWCNT-O and MWCNT-P had poor solubility in water, settled out of the suspension, and were not included in this analysis. All three MWCNT-F materials had a small particle size and high negative zeta potential values, consistent with increased stability of MWCNT-F in water. The polydispersity index (PDI) was relatively low showing relatively uniform particle size. The agglomerate size and zeta potentials were also measured in RPMI cell culture medium (Table 3), and the 5% Infasurf/saline vehicle (Table 4). In cell culture medium and Infasurf vehicle all agglomerates and zeta potentials were similar. The zeta potentials of the MWCNT were slightly more negative in Infasurf vehicle when compared to RPMI culture media. The endotoxin content of all MWCNT used in this study are found in Table 3.

**Table 2:** Particle size, zeta potential and polydispersibility index (PDI) of functionalized MWCNT in water

**MWCNT**	**Particle size**	**Zeta potential**	**PDI**
	**(nm)**	**(mV)**	
N/S-F	174	−43.4	0.35
W/S-F	160	−43.5	0.40
N/L-F	134	−43.8	0.34

N/S: narrow diameter/short length; W/S: wide diameter/short length; N/L: narrow diameter/long length.

F: functionalized MW.

**Table 3:** **Agglomerate size (mean ± range), zeta potential (mean ±** ***SD*), and endotoxin contamination of MWCNT in RPMI media**

**MWCNT**	**Hydrodynamic size**	**Zeta potential**	**E-toxin**	**E-toxin**
	**(nm)**	**(mV)**	**(EU/mg)**	**(ng/50 μg)**
N/S-O	568 ± 677	−10.27 ± .39	0.246	0.123
N/S-F	1845 ± 650	−9.77 ± .47	14.471	7.235
N/S-P	625 ± 131	−10.97 ± .75	0.299	0.149
W/S-O	810 ± 62	−9.73 ± 1.26	0.392	0.196
W/S-F	1161 ± 183	−9.73 ± .55	13.027	6.513
W/S-P	769 ± 52	−9.17 ± .80	11.193	5.596
N/L-O	396 ± 22	−10.6 ± .4	0.408	0.204
N/L-F	669 ± 136	−9.32 ± .16	2.629	1.31
N/L-P	441 ± 69	−10.93 ± .55	0.224	0.112

N/S: narrow diameter/short length; W/S: wide diameter/short length; N/L: narrow diameter/long length.

O: original MW; F: functionalized MW; P: purified MW.

**Table 4:** **Agglomerate size (mean ± range), and zeta potential (mean ±** ***SD*), of particles in 5% infasurf/saline**

**MWCNT**	**Hydrodynamic size (nm)**	**Zeta potential (mV)**
N/S-O	764 ± 210	−15.27 ± 1.86
N/S-F	1045 ± 496	−12.9 ± 1.0
N/S-P	1011 ± 411	−14.77 ± .59
W/S-O	867 ± 510	−14.4 ± 1.51
W/S-F	950 ± 158	−12.9 ± 1.48
W/S-P	945 ± 560	−12.77 ± .80
N/L-O	846 ± 219	−14.07 ± .64
N/L-F	1091 ± 263	−12.36 ± .64
N/L-P	861 ± 225	−14.03 ± .85

N/S: narrow diameter/short length; W/S: wide diameter/short length; N/L: narrow diameter/long length.

O: original MW; F: functionalized MW; P: purified MW.

### THP-1 Cytotoxicity and NLRP3 Inflammasome Activation

**Figure 3:**
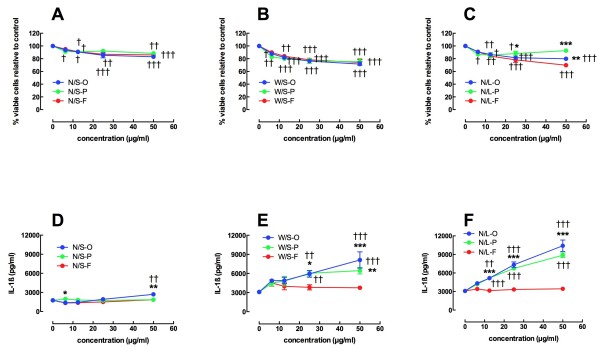
**Cell viability data and IL-1b release for THP-1 cells exposed to various MWCNT for 24 hours.** Data expressed as mean ± SEM percent viable cells compared to no particle control condition for **A** - **C**. Data expressed as mean ± SEM pg/ml IL-1β for **D** – **F. A**) Cell viability for the 3 variants of the narrow/short MWCNT. **B**) Cell viability for the 3 variants of the wide/short MWCNT. **C**) Cell viability for the 3 variants of the narrow/long MWCNT. **D**) IL-1β release for the 3 variants of the narrow/short MWCNT. **E**) IL-1β release for the 3 variants of the wide/short MWCNT. **F**) IL-1β release for the 3 variants of the narrow/long MWCNT. Asterisks indicate significance *** at P < 0.001, ** at P < 0.01, * at P < 0.05 compared to functionalized MWCNT variant at the same concentration. Daggers indicate significance ††† at P < 0.001, †† at P < 0.01, † at P < 0.05 compared to the 0 μg/ml no particle control.

The initial *in vitro* screening for bioactivity of individual MWCNT was done using human monocyte-like THP-1 cells transformed to a differentiated macrophage phenotype by vitamin D_3_ as described in Methods. This model has shown to be a reliable screening tool for detection of bioactive nanomaterials [6,25]. Figure 3A, C shows the 24 hr viability data for all particles tested separated by particle size in a dose range of 0, 6.25, 12.5, 25, and 50 μg/ml. All MWCNT showed significant cell death compared to no particle control or 0 μg/ml condition. However, there were no significant differences between specific doses of MWCNT-O, MWCNT-P, or MWCNT-F with the exception of the N/L-O, where the functionalized version was more toxic at the highest particle concentration (Figure 3C). The nominal decrease in viability was only about 10 percent at the highest concentration in these MWCNT. Compared to data reported for some other MWCNT samples [8,25], the cytotoxicity was relatively low.

Figure 3D, F shows data for IL-1β release from transformed THP-1 cells exposed to individual MWCNT for 24 hrs. Compared to the 0 μg/ml, no particle control, only the N/S-O at the highest concentration was significantly increased. There were a couple of significant differences between the variants at the lowest and highest particle concentrations (Figure 3D) with the N/S-F being significantly higher at 6.25 μg/ml and the N/S-O being significantly higher at 50 μg/ml. In contrast, when the diameter of the MWCNT was increased in the W/S materials, there was a significant increase in IL-1β release by the W/S-O and W/S-P MWCNT. However, the W/S-F material still showed no increase in IL-1β across concentrations, unlike the purified and original versions of this MWCNT (Figure 3E). Similar to the W/S material, the N/L MWCNT had increases in IL-1β with the original and purified, but not functionalized MWCNT (Figure 3F). The significance compared to functionalized MWCNT started at 12.5 μg/ml due to an enhanced IL-1β output relative to the W/S-O MWCNT.

### In Vitro C57BL/6 AM Cytotoxicty and NLRP3 Inflammasome Activation

**Figure 4:**
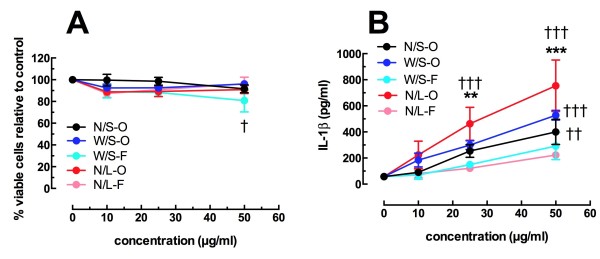
**Cell viability and IL-1β release data for C57BL/6 AM cells exposed to various MWCNT for 24 hours. A**) Mean ± SEM percent viable cells compared to no particle control condition. **B**) Mean ± SEM IL-1β as pg/ml. Asterisks indicate significance *** at P < 0.001, ** at P < 0.01 compared to both functionalized MWCNT variants at the same concentration. Daggers indicate significance ††† P < 0.001, †† at P < 0.01, † at P < 0.05 compared to the 0 μg/ml no particle control.

An abbreviated list of MWCNT was tested using AM isolated from C57Bl/6 mice. Since the original and purified materials of the different MWCNT were similar in bioactivity using the THP-1 cells the purified materials were eliminated from additional testing to minimize the use of mice. The N/S-F was also eliminated since it was not different from the N/S-O in the THP-1 experiments. Figure 4A shows the viability data using AM exposed to the remaining MWCNT for 24 hrs. With the exception of the W/S-F at the highest concentration, there were no significant decreases in viability compared to the 0 μg/ml, no particle control. In addition, there was no significant difference across concentrations for any specific MWCNT, effectively denoting these MWCNT as essentially nontoxic in this model at these concentrations (0, 10, 25, 50 μg/ml). The same five MWCNT were tested for IL-1β release in Figure 4B. Neither of the functionalized MWCNT was different from the no particle control. However, the three original materials showed dose dependent increases in IL-1β release. The N/L-O MWCNT was the only particle that deviated significantly from the others, as it was higher than the two functionalized MWCNT at 25 and 50 μg/ml. All of these AM results were consistent with the THP-1 results with the one exception that the N/S-O material produced significant IL-1β release compared to control. Taken together, the results indicate that increasing the diameter or the length of MWCNT increased the bioactivity of MWCNT and consistent with previous publications [11,25], where functionalized MWCNT were not significantly bioactive regardless of size.

### In Vitro TEM of 1.5 hr MWCNT uptake in C57BL/6 AM

**Figure 5:**
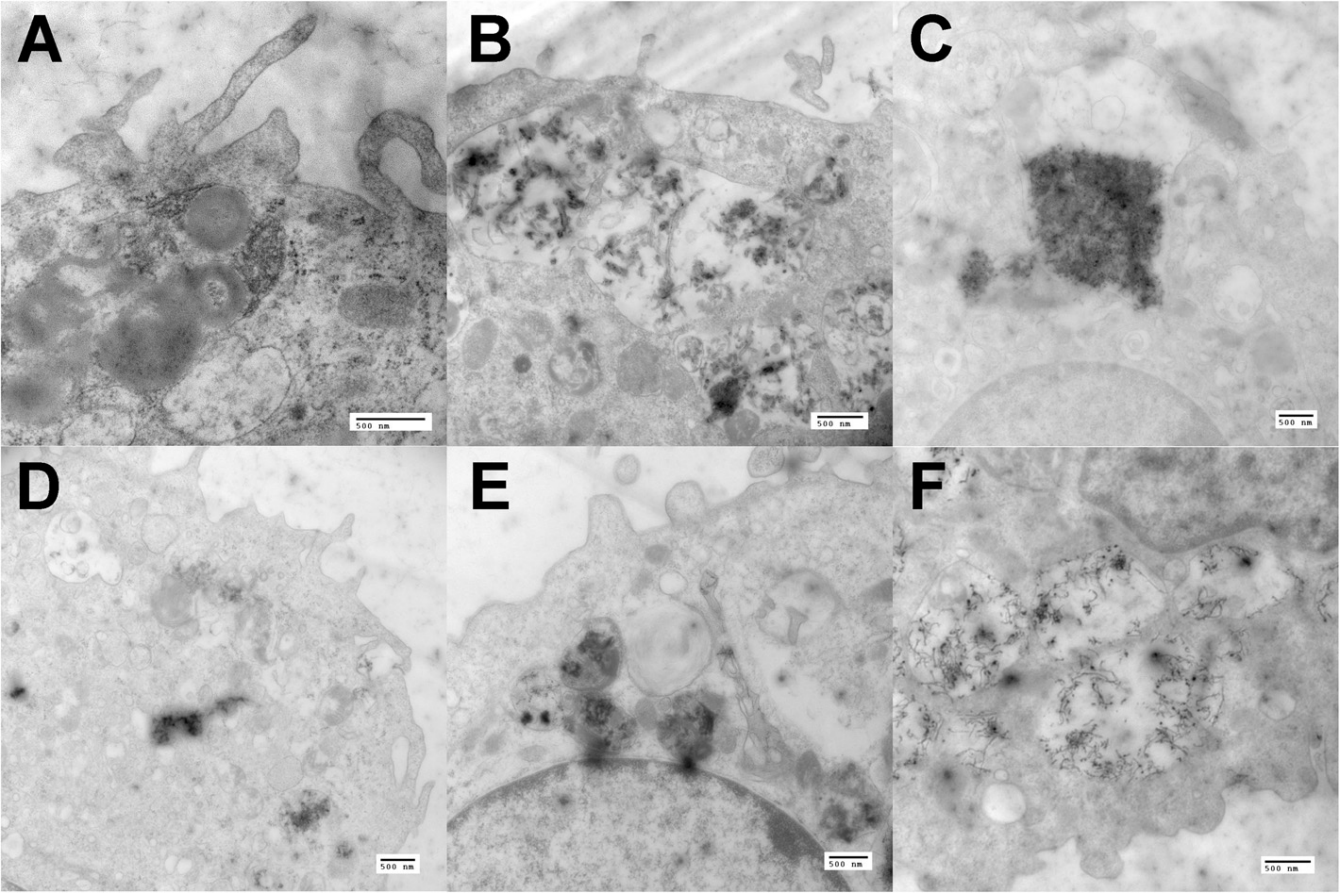
**High magnification TEM of MWCNT taken up by C57BL/6 alveolar macrophages 1.5 hr in vitro post-exposure (25 μg/ml). A**) N/S-O (narrow/short-original) MWCNT-exposed AM at 30 K x. **B**) N/S-F (narrow/short-functionalized) MWCNT-exposed AM at 20 K x. **C**) W/S-O (wide/short-original) MWCNT-exposed AM at 15 K x. **D**) W/S-F (wide/short-functionalized) MWCNT-exposed AM at 15 K x. **E**) N/L-O (narrow/long-original) MWCNT-exposed AM at 17 K x. **F**) N/L-F (narrow/long-functionalized) MWCNT-exposed AM at 20 K x. Black spotted/speckled areas indicate areas of organized phagosomal particle retention in the cytoplasm of the macrophage cell.

Electron microscopy was performed on isolated C57BL/6 AM as described in Methods to determine whether all the MWCNT were being internalized and to examine any possible differential process that might account for the differing effects seen in Figure 4. Figure 5A, F shows high magnification internalized MWCNT in the cytoplasm region of the AM cells. All the original and functionalized MWCNT variants used in this study were internalized (purified MWCNT uptake was not imaged). There were no obvious differences in the particle uptake, as it appeared that all of the MWCNT variants were internalized into organized phagosomal structures consistent with the early process involved with NLRP3 inflammasome activation.

### In Vivo C57BL/6 Exposure 24 hr Lavage Cell Count and Differential

**Figure 6:**
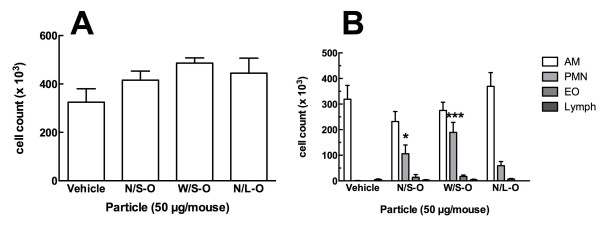
**Cell count and differential from lung lavage 24 hours post MWCNT instillation. A**) Mean ± SEM cell numbers for four instillation conditions. **B**) Cell differentials expressed as mean ± SEM cell numbers for retrieved cell types. AM – macrophages, PMN – neutrophils, EO – eosinophils, Lymph – lymphocytes. Asterisks indicate significance *** at P < 0.001, * at P < 0.05 compared to same cell type for Vehicle. n = 6 mice per condition.

The *in vitro* data demonstrated that the three MWCNT-O materials were all bioactive to varying degrees, so these three MWCNT materials were evaluated *in vivo* using C57Bl/6 mice exposed to 2 mg/Kg (50 μg/25 gm mouse) by oropharyngeal aspiration. Following lung lavage and cell count 24 hr post-exposure, the cells were cultured ex vivo ± LPS for an additional 24 hrs. A second group of mice was exposed for 7 days, and the lungs removed and sectioned for histological determination of pathology. Figure 6A shows the total cell count after 24 hr of exposure to the three MWCNT-O. There was no significant difference among the MWCNT-O exposures, although the cell count tended to be higher in the mice exposed to MWCNT-O regardless of size. Figure 6B shows the cell differentials at 24 hrs post exposure. All three MWCNT-O exposed groups showed increases in PMN compared to the vehicle-exposed group. The increases in PMN were statistically significant for N/S-O and W/S-O exposed mice. However, the N/L-O exposed group had a smaller increase in PMN compared to the other two MWCNT-exposed groups, but did not achieve statistical significance.

### Ex Vivo Cell Culture Cytokine Release

**Figure 7:**
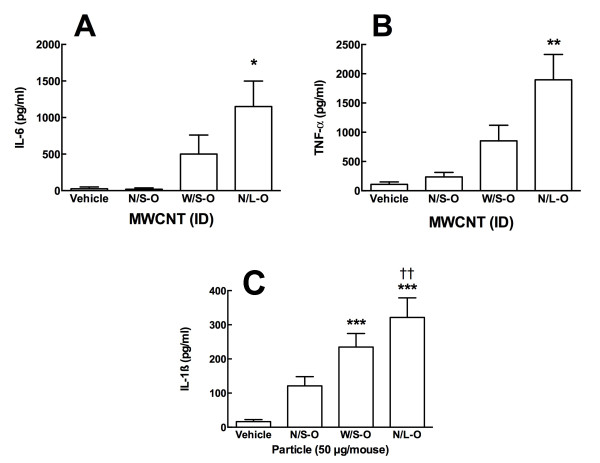
**Ex vivo cytokine release from cells recovered in lung lavage at 24 hours post MWCNT instillation. A**) IL-6 release from lavaged cells without LPS co-culture. **B**) TNF-α release from lavaged cells without LPS co-culture. **C**) IL-1β release from lavaged cells with LPS co-culture (20 ng/ml). Asterisks indicate significance *** at P < 0.001, ** at P < 0.01, * at P < 0.05 compared to Vehicle. Dagger indicates significance † at P < 0.05 compared to the original narrow/short MWCNT variant. n = 3 mice per condition.

The lavaged cells were cultured for 24 hr in the presence and absence of a small amount (20 ng/ml) LPS in order to determine the bioactivation state of AM *in vivo*. There appeared to be a hierarchy of IL-6 release among the three samples with N/S-O < W/S-O < N/L-O, although only the N/L-O material caused a significant release of IL-6 from the cells exposed to the material *in vivo* without LPS (Figure 7A). Likewise, Figure 7B shows the TNF-α release from the same samples not exposed to LPS. Similarly, the *in vivo* exposure of the N/L-O MWCNT was the only group to show significant increases in TNF-α although the pattern of AM TNF-α release among the groups was similar. Note: the LPS-exposed cells release copious amounts of IL-6 and TNF-α and this data is not presented. The LPS-exposed cell culture was mainly to assess NLRP3 inflammasome activated IL-1β release, as the LPS at this dose does not cause significant background IL-1β release in this culture model. Hence, Figure 7C shows the LPS co-stimulated IL-1β release in 24-hr culture. In this case, all particle exposures elicit a response with significant increases for W/S-O and N/L-O exposure *ex vivo*. Again, the cytokine release pattern of *in vivo* material-exposed AM was identical. Specifically, the N/L-O exposed group was significantly higher IL-1β than the N/S-O group. Taken together, the *ex vivo* cytokine release data indicates that the longer, larger MWCNT stimulates prolonged inflammatory cytokine release, which could play a major role in any developing lung pathology. Furthermore, the pattern of bioactivity *in vivo* (*ex vivo* assay) was identical to that observed *in vitro*.

### Lung Pathology After 7 Days Post MWCNT-exposure

**Figure 8:**
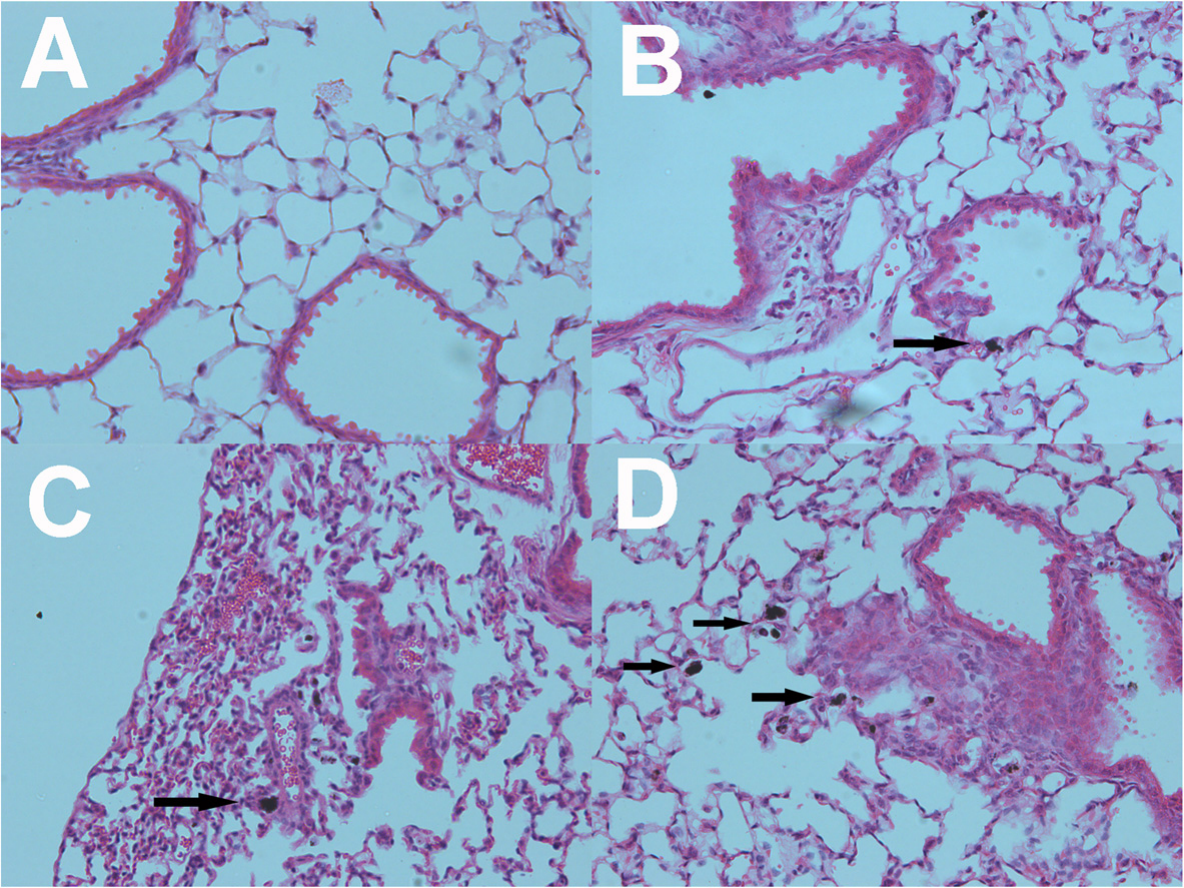
**Representative photomicrographs in bright-field microscopy of the four in vivo MWCNT instillation conditions at day 7. A**) vehicle-exposed. **B**) narrow/short-original MWCNT-exposed. **C**) wide/short-original MWCNT-exposed. **D**) narrow/long-original MWCNT-exposed. Black arrows indicate areas of particle retention. All images at 200x.

**Figure 9:**
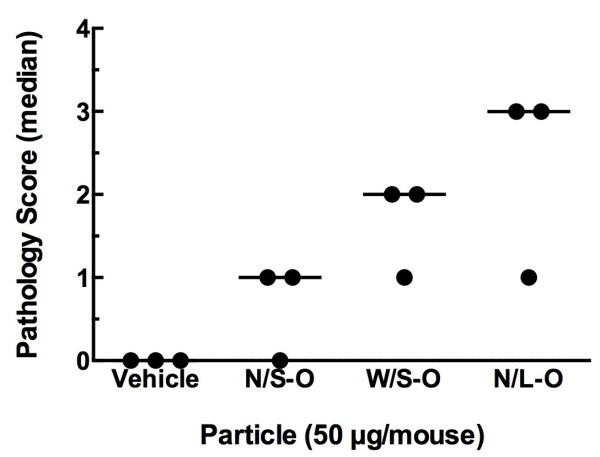
**Pathology scoring on a 5-point scale for lung sections at day 7.** A score of 0 indicates no deviation from normal. A score of 4 indicates the maximum deviation possible from normal. Data expressed as median pathology score with the dots representing individual scores. There was no statistical significance by non-parametric testing vs. a 0 score. n = 3 mice per condition.

As previously described, C57BL/6 mice were examined for histology and particle burden determination after 7-day MWCNT-O exposures. The lung pathology was scored on a subjective 5-point scale with 0 being ‘no deviation’ from normal lung and 4 being the ‘maximum deviation’ from normal lung histology. There was no distinction made between types of lung pathologies. Inflammation, increased cellularity, and fibrotic lesions were given the same weight of consideration. The pathology score simply reflects the degree of deviation from normal and how much of the lung section was affected. Figure 8A, D shows representative photomicrograph evidence of the three MWCNT-O plus vehicle exposure conditions. The black arrows indicate areas of obvious particle accumulation. The extra cellularity and inflammation was not necessarily correlated with the presence of particles. The summarized pathology scoring data is presented in Figure 9. Due to a lack of statistical power with a sample size of 3, the non-parametric analysis did not detect any significant differences compared to control. Nevertheless, the trend was consistent with the hypothesis that the larger MWCNT material caused more lung pathology. Taken together, these data were consistent with the previous *in vitro* and *in vivo* (*ex vivo*) bioactivity assessments.

### Particle burden after seven days post MWCNT-exposure

**Figure 10:**
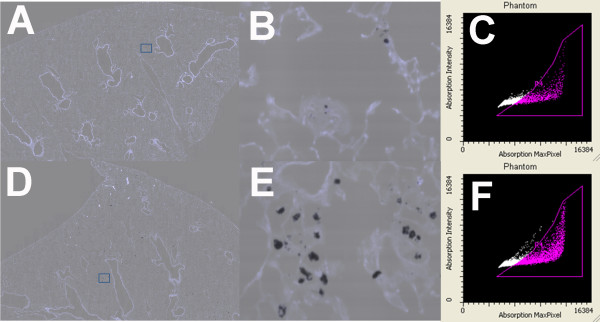
**Example of how MWCNT retention is determined in an unstained lung section. A**) Low resolution digitized image, composed of auto-fluorescence (tissue) and light loss (MWCNT), of lung section from N/S-O MWCNT-exposed mouse. Blue box indicates the scanning area. **B**) High resolution digitized image, composed of auto-fluorescence (tissue) and light loss (MWCNT), of scanning area from Figure 10A. **C**) Gated scatter-gram with total scan areas collected where the purple area indicates positive phantom contours for particle deposition. **D**) Low resolution digitized image, composed of auto-fluorescence (tissue) and light loss (MWCNT), of lung section from N/L-O MWCNT-exposed mouse. Blue box indicates the scanning area. **E**) High resolution digitized image, composed of auto-fluorescence (tissue) and light loss (MWCNT), of scanning area from Figure 10C. **F**) Gated scatter-gram with total scan areas collected where the purple area indicates positive phantom contours for particle deposition.

**Figure 11:**
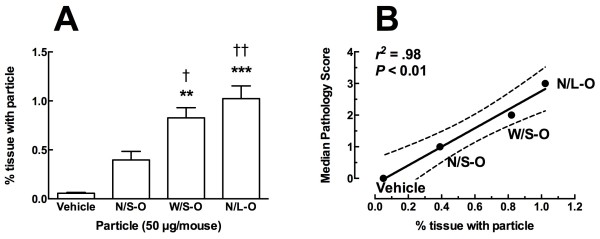
**Particle burden data at day 7 and the relationship to pathology. A**) Particle burden data for the four MWCNT instillation conditions at day 7*.* Data expressed as mean ± *SEM* percent tissue with particle. Asterisks indicate significance *** at *P* < 0.001, ** at *P* < 0.01 compared to Vehicle. Daggers indicate significance †† at *P* < 0.01, † at *P* < 0.05 compared to the original narrow/short MWCNT variant. **B**) Regression of day-7 particle retention verses day-7 pathology score for the MWCNT instillation conditions.

The same lung samples analyzed for pathology (different unstained sections of the same lobe) were used to determine the extent of particle retention in the lungs at 7 days. The lung sections in this case were unstained and processed as described in Methods. An example of how this analysis was obtained is shown in Figure 10. Figure 10A shows a low-resolution digitized image, of a lung section from a N/S-O-exposed mouse. The blue box indicates the scanning area shown as a high-resolution digitized image (Figure 10B). A few N/S-O MWCNT aggregations are evident by the black areas. In contrast, Figures 10D and 10E show the corresponding images for scanned lung sections of a N/L-O-exposed mouse. A clear increase in black areas is evident in Figure 10E indicating increased retention in the lung of these MWCNT compared to the smaller MWCNT. Figures 10C and 10F show the corresponding scattergrams with the particle positive areas indicated in the purple-gated area. Figure 11A shows the summary results of this iCys quantitation of particle retention in the lung tissue. Only the W/S-O and N/L-O MWCNT instillations resulted in significant particle retention compared to no particle control lungs, with the N/L-O having the most retention in a seven-day particle exposure. In addition, these two MWCNT-O instillations were also significantly increased over the particle retention in N/S-O-exposed lungs. Taken together with the pathology findings, this data demonstrates a positive significant correlation of particle retention regressed with pathology as shown in Figure 11B.

### The potential predictive value of the THP-1 model

**Figure 12:**
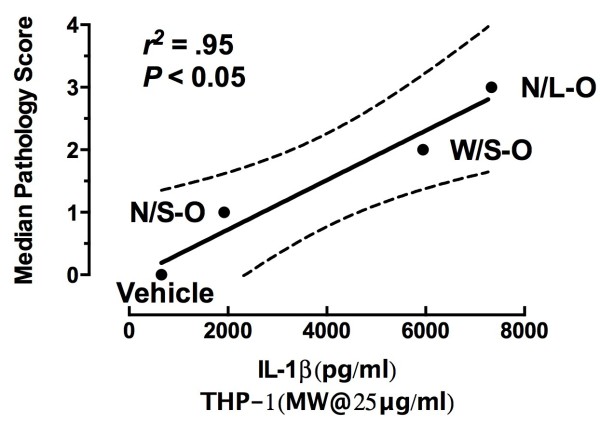
Regression of in vitro THP-1 IL-1β release at 25 μg/ml verses day-7 in vivo pathology scores for the MWCNT instillation conditions.

In order to demonstrate the validity of the *in vitro* THP-1 model as a valuable tool in revealing possible bioactive nanomaterials, a regression analysis of IL-1β release at 25 mg/ml in the THP-1 cells to pathology scores was done (Figure 12), using the THP-1 results as the predictor variable for lung disease. Note: several of the MWCNT concentrations worked in this regression model, but the 25 μg/ml was simply the best fit. The graph shows a positive significant relationship between THP-1 stimulation of IL-1β release at 24 hr with 7-day pathology. Therefore, the *in vitro* THP-1 data can be a potential predictor of the pathological outcome of MWCNT exposure at least in this mouse model. This is probably due to the importance of IL-1β in the initial and sustained inflammation in particle-exposed lungs [26].

## Discussion

Increased production of nanomaterials, especially carbon nanotubes, has raised concerns over potential risks of adverse human health effects since there is the likelihood of increasing exposures with time. However, even within one category of carbon nanotubes, such as multiwalled carbon nanotubes (MWCNT), there is a wide range of materials that vary with size, contaminants, functionalization, etc. Although a large number of studies have examined the biological responses of individual nanomaterials, there are fewer reports that examined the effect of changing properties of a series of materials on biological outcomes [27]. For example, a few studies have examined the influence of carboxylation of MWCNT or other surface functional modifications [25,28-30]. There have also been a few reports on the effect of length of MWCNT with respect to biological activity [3,9,12], suggesting that bioactivity increases with length. The current study examined length, diameter, as well as, purification and functionalization on bioactivity *in vitro* and *in vivo*.

The physical characterization of the different sets of MWCNT demonstrated that purification of the original samples effectively removed contaminants left over from the particle generation process (Table 1). Multiple forms of analysis demonstrated that the purified samples were all effectively carboxylated to similar extents. The carboxylation led to an apparent slight increase in diameter of each of the three original MWCNT (Table 1). As expected the original and purified samples all had poor solubility in water, falling rapidly out of suspension, while the functionalized materials all had high negative zeta potentials and increased stability in water (Table 2). However, when suspended in cell culture medium (RPMI) or instillation vehicle all 9 samples had roughly similar agglomerate sizes and very similar zeta potentials (Tables 3 and 4). Agglomerate particle size and zeta potential was not likely to account for any differences in the observed relative biological activity among any of the MWCNT samples. Therefore, this MWCNT library permitted the determination of biological activity comparing length, diameter, purity and carboxylation.

The potential mechanisms responsible for nanoparticle-induced lung inflammation and pathology are under active investigation in many laboratories. A pathway of current interest revolves around the ability of phagocytosed nanoparticles to cause phagolysosomal membrane permeability leading to release of cathepsin B into the cytoplasm, which has been implicated in the assembly of the NLRP3 inflammasome in macrophages. Macrophages are key innate immune cells regulating inflammation in many tissues including the lung [23,31,32]. The assembled inflammasome results in activation of Caspase-1 and cleavage of inactive pro-IL-1β into its active form. The mechanism of IL-1β release from macrophages is not completely understood, but IL-1β has been linked to both initial and sustained inflammation resulting from nanoparticle exposures *in vivo*[33]. The central issue regarding particle-induced inflammation relates to what property(ies) of nanoparticles (MWCNT), is most responsible for causing phagolysosomal membrane permeability.

The screening phase of the study utilized human THP-1 cells to initially evaluate all 9 of the MWCNT samples in a similar manner as previously described [6,30]. The advantages of using THP-1 cells are that they are relatively easy to grow, appear to be fairly predictive of *in vivo* outcomes and decrease the dependency on animal models. Dose response studies for all materials demonstrated, for the N/S and W/S MWCNT at least, that purification or functionalization did not affect toxicity. In contrast, for the long MWCNT purification decreased toxicity and functionalization slightly increased toxicity compared to the original material at the highest concentration tested. Nevertheless, toxicity was more dependent on concentration than any other property including length or diameter (Figure 3).

In contrast, bioactivity as measured by inflammasome-dependent IL-1β production was dependent on all parameters (concentration, diameter, length and functionalization). The N/S MWCNT demonstrated very little bioactivity. With an increase in either diameter or length there was a significant increase in bioactivity that was dose dependent (Figure 4). There was a slight reduction in bioactivity due to purification and a highly significant decrease as a result of functionalization, even though carboxylated MWCNT were still effectively taken up by macrophages *in vitro* and *in vivo* as demonstrated in other studies [25,29]. In order to confirm that length, diameter and functionalization did not affect uptake, TEM imaging was done with primary AM. The results confirmed that qualitatively there were no differences in the uptake of any of the materials tested confirming that the bioactivity was dependent on intracellular events rather than uptake. The rank order of bioactivity of the three original materials was N/S < < W/S < N/L. Surface area measurements of the three original MWCNT were made to determine how much variability existed and whether it could contribute to the differences in bioactivity. The surface areas were in the same order as the bioactivity. While this might appear to contribute to the relative ranking it would not explain why even relatively high doses of N/S caused much less IL-1β production than lower doses of the other two materials where the surface area for the N/S would have been equal or greater than the other two materials. Since purification did little to affect bioactivity, the remainder of the study focused on the three original and functionalized materials with the exception that the N/S-F was not examined due to the lack of any detectable toxicity or bioactivity in this sample. Regarding the diameter/length permutation that was not tested (W/L), one would hypothesize that this MWCNT would have as much or more bioactivity compared to the W/S and N/L MWCNT. Overall, the *in vitro* findings of purification and functionalization are consistent with previous studies [25,30].

*In vitro* studies were also conducted with primary AM isolated from C57BL/6 mice. Dose response examination of the toxicity to primary AM was similar to the THP-1 results with little toxicity evident and no significant differences comparing any of the groups. The outcome of the bioactivity (IL-1β release) with the primary AM was very similar to the THP-1 cells (Figures 3 and 4). The rank order of the original materials was N/S < W/S < N/L and significantly less bioactivity was observed for the two functionalized MWCNT. Overall, the results were highly comparable between THP-1 cells and primary AM supporting the notion that THP-1 cells can be used to screen the *in vitro* bioactivity of nanomaterials reducing the dependency on mice to obtain primary cells. It should be noted that three of the MWCNT samples (N/S-F, W/S-F and W/S-P) demonstrated slight endotoxin contamination (Table 3). However, based on our findings this small amount of endotoxin did not contribute to any false positives for the bioactivity of the MWCNT (data not shown). The functionalized MWCNT had little bioactivity and the purified samples were less active than the original. Since the functionalized MWCNT demonstrated little bioactivity they were not used for the *in vivo* studies.

The *in vivo* studies focused on two time points, 24 hours and 7 days. At 24 hours there was no difference in the inflammatory response (PMN increase) among the three original MWCNT (Figure 6). This outcome was not unexpected since such a short time point did not appear to be highly discriminatory. Similar results were obtained in previous studies where initial responses to any material indicated an overall inflammatory response that became more discriminatory with time [18,28]. Furthermore, the *ex vivo* component of this study, using lavaged cells obtained at 24 hr post MWCNT delivery, subsequently cultured for 24 hr, was highly consistent with the *in vitro* component (Figure 7). There was an identical pattern for IL-1β, IL-6 and TNF-α production (N/S < W/S < N/L) even though all mice received the same mass of MWCNT. Consequently, the *ex vivo* results mirrored the *in vitro* studies for both primary AM and THP-1 cells. It should also be noted that the absolute amount of IL-1β produced was similar to that observed *in vitro* (Figure 4B).

The 7-day study examined the relative inflammation/pathology of the lungs and retention of MWCNT (Figures 8 and 9). The pattern of outcome was N/S < W/S < N/L, which was consistent with the *in vitro* and *ex vivo* results. We also examined lung retention of the three MWCNT at 7 days using a unique technique by Laser Scanning Cytometry as described in the Methods. The results clearly indicated that increasing width or length of the MWCNT increased retention (decreased clearance). Furthermore, the pattern was the same for tissue retention as for bioactivity. Therefore, the relationship between lung tissue retention and lung pathology was highly significant (Figure 11), indicating lung particle burden is an important component of pathology.

The overall outcome of the study is that the *in vitro* results using THP-1 cells were highly predictive of the *in vivo* 7-day lung pathology (Figure 12). These findings are also consistent with our previous studies, supporting the notion that using THP-1 cell release of IL-1β can predict the rank order of bioactivity of a series of nanomaterials [6,30]. Studies with THP-1 cells can be done quickly and cost effectively. In addition, measuring IL-1β production is highly consistent with the proposed importance of NLRP3 inflammasome activity in the resultant chronic inflammation and pathology predicted for various particles [8,23,28,34]. In contrast, toxicity did not appear to be a distinguishing feature among any of the MWCNT samples and was not predictive of the differences in pathology. Furthermore, IL-1β is mechanistically linked to chronic inflammation and further supports the notion that IL-1β production from THP-1 cells can be used as an initial screening tool.

Thus far the MWCNT sizes have only been considered in the context of individual tubes. However, because of the tangled nature of the MWCNT used in this study the suspensions are agglomerates of many individual tubes. Since the agglomerate size and other physical properties of the three original nanomaterials were roughly similar, an explanation for the rank order of bioactivity may not be readily apparent when considering the delivered tubes compared to individual tubes. For tangled MWCNT in agglomerates a simple consideration of high aspect ratios does not seem to apply. All three of the MWCNT as individual fibers have high aspect ratios, but more importantly they form similar size agglomerates that are taken up by the macrophages. As agglomerates there is no dimension that is long, but they are relatively large. Since the three MWCNT are similar with respect to surface chemistry their ability to either generate or scavenge reactive oxygen species would likely be similar [35]. The bioactivity of the different MWCNT would appear to be dependent on their ability to cause permeabilization of the phagolysosome, which precedes the release of cathepsin B leading to inflammasome assembly [31]. A possible explanation is that increasing the diameter (W/S-O compared to N/S-O) would make the W/S-O MWCNT more rigid increasing the potential of a physical/mechanical mechanism interacting with the phagolysosomal membrane, which is coincidently consistent with observations from a recent lung cancer study [36]. In a related manner the N/L-O would have more long dangling ends in the agglomerate resulting in more degrees of freedom/more mobility to interact with the phagolysosomal membrane surface increasing the probability of interactions resulting in permeabilization. In this manner, the results from this study fit well into the proposal that the MWCNT bioactivity would be dependent on the ability of the particles to cause permeabilization of the phagolysosome.

The overall findings, that increases in diameter or length increase the bioactivity, are consistent with previous studies showing that longer MWCNT were more bioactive [2,3,9,12]. Although some concerns were expressed [12], that some of the bioactivity might be due to artifacts of preparation resulting in more reactive ends, this would not be consistent with our findings since the short MWCNT, with a much higher concentration of “ends”, had minimal bioactivity. Taking all of the physical characterization into consideration it would appear that the increased bioactivity was due to an increase in diameter or length and not some other feature of surface chemistry.

The materials used in this study were less active than other MWCNT that we have examined and likely due to the low level of metal contaminants in the original materials [8]. Nevertheless, the results from the functionalization are consistent with earlier publications demonstrating that -COOH functionalization decreases both toxicity and bioactivity [25,28-30]. Furthermore, the results indicate that regardless of size, carboxylation decreases bioactivity. The explanation for -COOH functionalization to decrease bioactivity is not clear. Based on the above speculation regarding the influence of width and length it also may be related into how the –COOH groups interact with the membrane surface of the phagolysosome. It could also be that carboxylation protects against MWCNT permeabilization of phagolysosomal membranes upstream of inflammasome activation.

## Conclusions

We have demonstrated that increasing the diameter or length of MWCNT significantly increases NLRP3 inflammasome driven IL-1β production *in vitro* and inflammation/pathology *in vivo*. These results imply that structural differences due to changes in diameter or length are important in defining interactions of the MWCNT with phagolysosomal membranes. In addition, carboxylation of the MWCNT was sufficient to significantly decrease the bioactivity of MWCNT *in vitro*, suggesting that not only shape and size, but also surface properties of MWCNT influence bioactivity of nanomaterials. This information will be important in helping design safer nanomaterials.

## Methods

### Preparation of functionalized MWCNT and purified MWCNT

This study used three types of original, “as-received” MWCNT namely narrow diameter and short length (10–20 nm, 0.5-2 μm) referred to as MWCNT-N/S, narrow diameter and long length (10–20 nm, 10–30 μm) referred to as MWCNT-N/L, and large diameter and short length referred to as MWCNT-W/S (30–50 nm, 0.5-2 μm). All MWCNT were acquired (these original materials are referred to as MWCNT-O) from Cheaptubes Inc. prior to being purified and functionalized. The synthesis of the functionalized MWCNT (MWCNT-F) and purified MWCNT (MWCNT-P) were carried out under the Microwave Accelerated Reaction System using methods described before [37-39]. For purification of MWCNTs, as obtained MWCNT-O were added to the reaction chamber together with dilute 1 M HNO_3_. The reaction vessels were subject to microwave radiation at a preset temperature of 100°C for 10 min. After cooling to room temperature, the product was vacuum filtered on a 10 μm filter using Milli-Q water, until the filtrate reached a neutral pH. The sample was then dried in a vacuum oven at 65°C until constant weight. For MWCNT-F preparation, a pre-weighed amount of MWCNT was added to reaction chamber together with a mixture of concentrated 1:1 H_2_SO_4_ (95%-98%) and HNO_3_ (70%). The reaction vessels were subject to microwave radiation at a preset temperature of 140°C for 20 min. After cooling to room temperature, the product was vacuum filtered using Milli-Q water with pore size 10 μm, until the filtration reached a neutral pH, was dried in a vacuum oven at 70°C until constant weight. In the rest of the manuscript the individual MWCNT are defined as, N/S-O, N/S-P, N/S-F, etc., unless they are being discussed as a general category, e.g., MWCNT-F.

### MWCNT Characterization

The samples prepared in Milli-Q water were characterized using a scanning electron microscope (SEM, LEO 1530 VP) equipped with an energy-dispersive X-ray analyzer (EDX) (Carl Zeiss, Oberkochen, Germany). The length and diameter were also measured using SEM. These were measured by averaging at least 50 samples. The Fourier Transformed Infrared spectroscopy (FTIR) spectra were taken using a PerkinElmer instrument where the MWCNT samples were mixed with purified potassium bromide (KBr) then pressed into pellets (PerkinElmer, Santa Clara, CA). Particle size and zeta potential were measured at 25°C using a Malvern Zetasizer nano ZS90 (Malvern Instruments, Worcestershire, UK) at a 90° detector angle. Specific surface areas of original MWCNT samples were measured using Quantachrome NOVA 3000 series (Model N32-11) High Speed Gas Sorption Analyzer (Boynton Beach, FL) at 77.40 K. Before each experiment, the samples were heated at 300°C and degassed at this temperature until constant vacuum for three hours. Endotoxin contamination was determined by washing/vortexing 1 mg/ml MWCNT in endotoxin-free water for 30 min followed by centrifugation at 16,000 × *g* for 15 min prior to assay. The assay (ToxinSensor) was performed according to the manufacturer’s protocol (GenScript, Piscataway, NJ).

### Experimental procedures

*MWCNT suspensions*:

All nanotubes were weighed and suspended in 5% Infasurf (calfactant, ONY, Inc. Amherst, New York) diluted in sterile saline. Nanotube suspensions were sonicated for 2 min at half max power in a Masonix cup-horn sonicator (XL2020, Farmingdale, NY) attached to a Forma circulating water-bath at 550 watts and 20 Hz (8000 Joules) at a stock concentration of 5 mg/ml.

### Human THP-1 cell line culturing

THP-1 cells, a human monocytic cell line obtained from ATCC, were suspended in RPMI media (MediaTech, Manassas, VA) supplemented with 10% fetal bovine serum, 50 μM beta-mercapto ethanol, 1 mM sodium pyruvate, 250 ng/ml amphotericin B, and 100 U/ml penicillin and streptomycin (all supplements Media Tech, Manassas, VA) in 75 cm^2^ flasks at 37°C. The cells in suspension were differentiated into a macrophage-like cell by adding 150 nM Vitamin D_3_ (1α, 25-dihydroxy, EMD Millipore, Darmstadt, Germany) for 24 hr. The semi-adherent cells were scrapped with a rubber policeman in the existing media (Corning, Corning, NY). The cells were then centrifuged at 400 × *g* for 5 min, the resulting cell pellet was re-suspended in 1 ml of complete media, and a 40 μl sample was then counted on a Z2 Coulter Counter (Beckman Coulter, Miami, FL). The cells were suspended at 1 × 10^6^ cells/ml and a small amount of phorbol 12-myristate 13-acetate (5 nM PMA, Sigma) and lipopolysacharride (10 ng/ml LPS, Sigma) was added. PMA co-stimulation is necessary to stimulate aggressive phagocytosis of the MWCNT. LPS co-stimulation is necessary to induce NF-κB translocation leading to pro-IL-1β synthesis for the NLRP3 inflammasome to cleave for IL-1β release in the transformed THP-1 model [3,34]. Cells, at a volume of 350 μl, were then pipetted in to 1.5 ml microfuge tubes. The MWCNT conditions were added from 5 mg/ml concentrated stock suspensions to the cells at a final concentration of 25 μg/ml. The MWCNT variants used a range of concentrations (0, 6.25, 12.5, 25, and 50 μg/ml). The resulting cell/particle suspension was mixed by pipette action. The cells were then transferred to 96-well tissue culture plates at 100 μl per well in triplicate (100 × 10^3^ cells/well), and cultured for an additional 24 hr. All cultures were maintained in 37°C water-jacketed CO_2_ incubators (ThermoForma, Houston, TX). Viability and IL-1β levels were determined as described below. Three experimental replicates were done for each experiment.

*Animals*: C57Bl/6 (2-months old, male) were housed in controlled environmental conditions (22 ± 2°C; 30-40% humidity, 12-h light: 12-h dark cycle) and provided food and water *ad libitum*. All procedures were performed under protocols approved by the IACUC of the University of Montana.

*Alveolar macrophage isolation:* Mice were euthanized by sodium pentobarbital (Euthasol™ Schering-Plough, Lot# 1JRR11V), and the lungs with the heart were removed. Lung lavage was performed using ice-cold PBS (pH 7.4). Lung lavage cells were isolated by centrifugation (400 × *g*, 5 min, 4°C) and cell counts obtained using a Coulter Z2 particle counter (Beckman Coulter, Miami, FL).

*Cell culture*: Alveolar macrophages (AM) cells were suspended in RPMI media supplemented with 10% fetal bovine serum, 0.05 mM 2-mercaptoethanol, sodium pyruvate, and supplemented with an antimycotic/antibiotic cocktail (Mediatech, Manassas, VA). Cells were suspended at 1 × 10^6^ cells per ml and then lipopolysaccharide (LPS, Sigma, St Louis, MO) at 20 ng/ml was added to stimulate pro-IL-1β formation. A 100 μl sample (100,000 cells) of cells were exposed to each MWCNT (ex: high dose 50 μg/ml equivalent to 5 μg/10^5^ cells equivalent to 15.62 μg/cm^2^ (5 μg on .32 cm^2^)) and experiments were conducted in 96-well plates for 24 h in 37°C water-jacketed CO_2_ incubators (ThermoForma, Houston, TX). Particle concentrations ranged from 0, 10, 25, 50 μg/ml. Media was collected for IL-1β assay and cell viability was determined by MTS assay. Ten to 12 mouse lung lavage collections were pooled, and this experiment was replicated three times.

### Toxicity assay

Cell viability was determined by MTS using the CellTiter^96^ assay (Promega, Madison, WI) according to the manufacturer’s protocol with a modification as described below. This assay used a colorimetric dye read by a colorimetric plate reader (Molecular Devices, Sunnyvale, CA). In order to avoid artifacts in the optical density values steps were taken to remove the MTS reagent (transferring it into another plate) from the cell/particle mixture adhered to the plate bottom. The formation of bubbles was avoided and the plate was read at 490 nm.

### Cytokine assays

Mouse and human IL-1β DuoSets were obtained from R&D Systems (Minneapolis, MN) and ELISA assays performed according to the manufacturer’s protocol. IL-6 and TNF-α DuoSet ELISA’s were also obtained from R & D Systems. Plates were read at 450 nm and data expressed as pg/ml.

### Electron microscopy

Isolated AM from C57BL/6 mice were exposed to MWCNT at 25 μg/ml for 1.5 h in suspension culture using 1.5 ml polypropylene tubes on a slowly rotating mixer (LabQuake Shaker, Lab Industries, Berkley, CA). The cells were washed once in PBS and resulting macrophage suspensions were fixed in 2.5% EM grade glutaraldehyde in cacodylate buffer at pH 7.2 (EMS, Electron Microscopy Sciences, Hatfield, PA). The cells were then rinsed in dH_2_O and resuspended in 1% osmium tetroxide (EMS) for 1 h and rinsed in dH_2_O. The cells were dried in a graded ethanol series followed by embedding of the cell pellet in epoxy resin. Thin sections were stained with 2% uranyl acetate (EMS) for 30 min at room temperature, rinsed in dH_2_O, and stained for 5 min with Reynolds lead citrate stain (EMS). The cells were imaged in a Hitachi H-7100 transmission electron microscope (Chula Vista, CA) at 75 kV.

### In vivo mouse exposures

All nanoparticles were suspended in 5% Infasurf (vehicle, 95% saline) as described above. Mice were exposed to nanoparticles by oropharyngeal aspiration. Briefly, the mice were anesthetized using inhalation isoflurane and a volume of 25 μl of particle suspension (2 mg/Kg or 50 μg/ 25 g mouse) was delivered into the back of the throat. By holding the tongue to the side, the solution was aspirated into the lungs. After 1 day the lungs were removed from a subset of exposed mice and lavaged with cold PBS as described above with the exception that individual mouse lavages were kept separate (no pooled samples). The isolated cells from these samples were cultured with and without LPS (20 ng/ml) for an additional 24 hours in a manor described above. The media was isolated and assayed for IL-1β, IL-6 and TNF-α. Cell differentials were determined by centrifuging a small sample (35 × 10^3^ cells) on to positively charged glass slides in a cytocentrifuge at 1500 rpm for 5 min (Shandon Cytospin 3, Thermo Fisher, Houston, TX). The slides were then stained in a Hematek slide stainer (Bayer Diagnostics, Dublin, Ireland) with a modified Wrigh-Giemsa stain (Protocol, Fisher, Houston, TX). The slides were allowed to dry. Differentials were conducted on a Zeiss microscope at 400× and 200 cell counts per slide. After 7 days a different subset of exposed mice were euthanized by sodium pentobarbital (Euthasol™), and the lungs were removed for fixation and histological examination. Six mice were used for each particle exposure condition.

### Histology

The lungs from each mouse were inflation-fixed through the trachea with 3% paraformaldehyde-PBS and submerged in the same fixative overnight at 4°C. The lungs were washed with cold PBS, dehydrated, and embedded in paraffin. Tissue sections (7 μm) were stained with hematoxylin-eosin (RAS Harris Hematoxylin and Shandon Alcohol Eosin) for histological analysis using a Thermo Shandon automated stainer (Shandon).

### Microscopy and pathology scoring

Mouse lung tissue sections were imaged at 100× using a Zeiss Axioskop attached to Zeiss digital camera and processed using Zeiss AxioVision software. An experienced observer scored the degree of lung disease visible in the lung sections using a 5 point scale (0, 1, 2, 3 and 4) with zero being no effect and 4 being extreme lung pathology evident. There were 3 exposed mice per MWCNT condition. The values shown in this paper are the median value for each condition.

### Cytometry technique for assessing lung particle burden

Fixed lungs (described above) were embedded in paraffin and sectioned on a microtome (7 um), then de-paraffinized and mounted on glass slides (unstained) and cover-slipped. A tissue analysis protocol was set up on a iCys CompuCyte Laser Scanning Cytometer (Cambridge, MA) to first conduct a low resolution scan of the entire tissue section using a 20× objective, 10 μm x-steps, a 488 nm laser and a 530/30 band pass filter to measure auto-fluorescence. The resulting tiled image showed the structure and outline of the tissue. The operator then hand-drew a region of interest around the area of tissue to further evaluate. Using the lobes of one side of the lung was sufficient to provide statistical representation of the entire lung. Once the regions were outlined, the slide was then re-scanned using a 40× objective and a much smaller x-step (0.25 um). Fluorescent and light-loss signals were detected and quantified by the photomultiplier tube (PMT) or photo-diode detectors for each field. The software converted the digitized signals into images that showed the delicate interstitial structure of the lung. Particles (or more likely, clumps of particles) showed up as black spots and were quantified using the light-loss detector (or scatter) to measure. “Phantom” contours were used to divide up the tissue into very small (8 um diameter) circles. If a contour showed light loss over background (threshold is set by observation on a test scan) it was counted as a particle-positive event, in addition quantified by how much light loss (intensity). A ratio of positive contours over total tissue contours was derived to compare one tissue to the next. Auto-fluorescence was used to define tissue and open airways or tears in the tissue were excluded from the total tissue contours to normalize differences from one section to the next. Seven non-serial sections were analyzed for each lobe and averaged. There were three mice per exposure condition.

### Statistical analyses

Statistical analyses involved comparison of means using a one or two-way *ANOVA* followed by Dunnett’s test or Bonferroni’s test to compensate for increased type I error. Ordinal level pathology score data was analyzed using Kruskal-Wallis followed by Dunn’s test. All probabilities were two-tailed unless otherwise stated. Statistical power was greater than 0.8. Linear regression analysis was performed to determine possible predictive relationships between variables. The strength of the relationship is expressed as the coefficient of determination (*r*^*2*^), indicating the proportion of variability in *X* explained by *Y*. Statistical significance was defined as a probability of type I error occurring at less than 5% (*P* < 0.05). The minimum number of experimental replications was 3. Graphics and analyses were performed on PRISM 5.0 and SPSS 20.0.

## Abbreviations

MWCNT: Multi-walled carbon nanotube; O: Original; P: Purified; F: Functionalized; N/S: Narrow/short; W/S: Wide/short; N/L: Narrow/long; NLRP3: NACHT, LRR and PYD domains-containing protein 3; AM: Alveolar macrophage; SEM: Scanning electron microscopy; FTIR: Fourier Transform Infrared Spectroscopy; DLS: Dynamic light scattering; PDI: Polydispersibility index; IL: Interleukin; LPS: Lipopolysaccharide; PMN: Polymorphonuclear; TNF: Tumor necrosis factor; EDX: Energy-dispersive X-ray; Hz: Hertz; MTS: (3-(4,5-dimethylthiazol-2-yl)-5-(3-carboxymethoxyphenyl)-2-(4-sulfophenyl)-2H-tetrazolium); PBS: Phosphate-buffered saline; PMA: Phorbol-12-myristate-13-acetate; KBr: Potassium bromide; PMT: Photomultiplier tube.

## Competing interests

The authors declare that they have no competing interests.

## Authors’ contributions

SM and ZW were responsible for the preparation and characterization of all nanomaterial samples. AH and RH conceived and participated in the design of the bioactivity studies in the work. RH was responsible for the data analysis. PS developed the assay for quantitation of MWCNT. RH and AH drafted the manuscript and all authors were involved in critical review. All authors read and approved the final manuscript.
